# Measuring health inequalities in the context of sustainable development goals

**DOI:** 10.2471/BLT.18.210401

**Published:** 2018-06-28

**Authors:** Ahmad Reza Hosseinpoor, Nicole Bergen, Anne Schlotheuber, John Grove

**Affiliations:** a Department of Information, Evidence and Research, World Health Organization, avenue Appia 20, 1211 Geneva 27, Switzerland.; b Faculty of Health Sciences, University of Ottawa, Ottawa, Canada.

## Abstract

Transforming our world: the 2030 agenda for sustainable development promotes the improvement of health equity, which entails ongoing monitoring of health inequalities. The World Health Organization has developed a multistep approach to health inequality monitoring consisting of: (i) determining the scope of monitoring; (ii) obtaining data; (iii) analysing data; (iv) reporting results; and (v) implementing changes. Technical considerations at each step have implications for the results and conclusions of monitoring and subsequent remedial actions. This paper presents some technical considerations for developing or strengthening health inequality monitoring, with the aim of encouraging more robust, systematic and transparent practices. We discuss key aspects of measuring health inequalities that are relevant to steps (i) and (iii). We highlight considerations related to the selection, measurement and categorization of dimensions of health inequality, as well as disaggregation of health data and calculation of summary measures of inequality. Inequality monitoring is linked to health and non-health aspects of the 2030 agenda for sustainable development, and strong health inequality monitoring practices can help to inform equity-oriented policy directives.

## Résumé

Transformer notre monde: le programme de développement durable à l'horizon 2030 promeut l'amélioration de l'équité en santé, ce qui implique un suivi continu des inégalités en matière de santé. L'Organisation mondiale de la Santé a élaboré une approche pour le suivi des inégalités en santé qui comprend plusieurs étapes: (i) déterminer la portée du suivi; (ii) collecter des données; (iii) analyser les données; (iv) communiquer les résultats; et (v) mettre en œuvre des changements. À chaque étape, des considérations techniques ont des conséquences sur les résultats et les conclusions du suivi et sur les mesures correctives qui en résultent. Ce document présente certaines considérations techniques pour le développement ou le renforcement du suivi des inégalités en santé, dans l'objectif d'encourager des pratiques plus fiables, plus systématiques et plus transparentes. Nous examinons des aspects clés de la mesure des inégalités en santé à prendre en compte dans les étapes (i) et (iii). Nous mettons en avant des considérations en lien avec la sélection, la mesure et la catégorisation des dimensions des inégalités en matière de santé, ainsi que la ventilation des données sur la santé et le calcul de mesures synthétiques des inégalités. Le suivi des inégalités est lié à des aspects sanitaires et non sanitaires du Programme de développement durable à l'horizon 2030, et des pratiques rigoureuses de suivi des inégalités en matière de santé peuvent contribuer à éclairer les directives axées sur l'équité.

## Resumen

Transformar nuestro mundo: el plan de 2030 para el desarrollo sostenible promueve la mejora de la igualdad en la salud, lo que implica un seguimiento continuo de las desigualdades en salud. La Organización Mundial de la Salud ha desarrollado un enfoque de múltiples pasos para el seguimiento de la desigualdad en la salud que consiste en: (i) determinar el alcance del seguimiento; (ii) obtener datos; (iii) analizar los datos; (iv) informar sobre los resultados; e (v) implementar los cambios. Las consideraciones técnicas de cada paso tienen implicaciones para los resultados y las conclusiones del seguimiento y las acciones correctivas subsiguientes. En este documento se presentan algunas consideraciones técnicas para desarrollar o fortalecer el seguimiento de la desigualdad en la salud, con el fin de fomentar prácticas más robustas, sistemáticas y transparentes. Se analizan aspectos clave de la medición de las desigualdades en la salud relevantes para los pasos (i) y (iii). Se destacan las consideraciones relacionadas con la selección, la medición y la categorización de las dimensiones de la desigualdad en la salud, así como la desagregación de los datos de salud y el cálculo de medidas sintetizadas de desigualdad. El seguimiento de la desigualdad está vinculado a los aspectos sanitarios y no sanitarios del programa de desarrollo sostenible de 2030, y unas prácticas rigurosas de seguimiento de la desigualdad en la salud pueden ayudar a fundamentar las directrices políticas orientadas a la igualdad.

## ملخص

تحويل عالمناترتقي *خطة التنمية المستدامة لعام 2030 * بتحسين العدالة الصحية، وهو ما يستلزم المتابعة المستمرة لأوجه عدم المساواة الصحية. طورت منظمة الصحة العالمية نهجًا متعدد الخطوات لرصد عدم المساواة في الصحة يتكون من: (1) تحديد نطاق المراقبة؛ و(2) الحصول على البيانات؛ و(3) تحليل البيانات؛ و(4) الإبلاغ عن النتائج؛ و(5) تنفيذ التغييرات. يكون للاعتبارات التقنية في كل خطوة آثار على نتائج واستنتاجات الرصد والإجراءات التصحيحية اللاحقة. تعرض هذه الورقة بعض الاعتبارات التقنية لتطوير أو تعزيز مراقبة عدم المساواة الصحية، بهدف تشجيع ممارسات أكثر قوة ومنهجية وشفافية. نحن نناقش الجوانب الرئيسية لقياس عدم المساواة الصحية ذات الصلة بالخطوتين (1) و(3). نحن نبرز الاعتبارات المتعلقة باختيار وقياس وتصنيف أبعاد عدم المساواة الصحية، وكذلك تحليل البيانات الصحية وحساب المقاييس الموجزة لعدم المساواة. يرتبط رصد عدم المساواة بالجوانب الصحية وغير الصحية لجدول أعمال التنمية المستدامة لعام 2030، ويمكن أن تساعد ممارسات مراقبة عدم المساواة الصحية القوية في توجيه لوائح السياسات الموجهة نحو المساواة.

## 摘要

《改变我们的世界——2030 年可持续发展议程》旨在改善健康不平等状况，这意味着该议程将对健康不平等进行持续监测。世界卫生组织推出了一种多步骤方法以监测健康不平等，步骤包括：(i) 确定监测范围；(ii) 获取数据；(iii) 分析数据；(iv) 汇报结果；以及 (v) 实施改变。每一项步骤的技术考量都会影响到监测的结果、结论及随后的补救措施。出于鼓励更多稳健性、系统性和透明性措施之目的，本文将会为发展和加强健康不平等的监测提供一些技术考量。我们对度量健康不平等过程中的关键部分即步骤 (i) 和 (iii) 进行讨论。我们重点关注与健康不平等维度的选择、度量及分类相关的考量，以及分解健康数据和计算不平等的概括性措施。不平等监测关乎 2030 年可持续发展议程中的健康和非健康领域，强有力的健康不平等监测措施有助于形成平等导向的政策指令。

## Резюме

Документ *Преобразование нашего мира: повестка дня в области устойчивого развития на период до 2030 года* содействует улучшению ситуации в обеспечении равенства в вопросах здравоохранения, что влечет за собой постоянный мониторинг неравенства в этих вопросах. Всемирная организация здравоохранения разработала многоэтапный подход к мониторингу неравенства в вопросах здравоохранения, состоящий из следующих этапов: (i) определения сферы мониторинга, (ii) получения данных, (iii) анализа данных, (iv) отчетности и (v) внедрения изменений. Технический анализ на каждом этапе имеет значение для результатов и выводов мониторинга и последующих корректирующих действий. В настоящем документе представлены некоторые результаты технического анализа по разработке или укреплению мониторинга неравенства в вопросах здравоохранения с целью поощрения более надежных, систематических и прозрачных практических действий. В статье обсуждаются ключевые аспекты определения неравенства в вопросах здравоохранения, которые имеют отношение к этапам (i) и (iii). Особое внимание уделено технологическому анализу, связанному с отбором, определением и категоризацией аспектов неравенства в вопросах здравоохранения, а также дезагрегированием данных о состоянии здоровья и расчетом сводных показателей неравенства. Мониторинг неравенства связан как с теми аспектами повестки дня по вопросам устойчивого развития до 2030 года, которые относятся к здравоохранению, так и с теми, которые не связаны со здравоохранением, поэтому четкая и последовательная практика мониторинга неравенства в вопросах здравоохранения может помочь в разработке директив, ориентированных на обеспечение равенства.

## Introduction

In 2015, 193 nations committed to the United Nations sustainable development goals (SDGs), defined in *Transforming our world: the 2030 agenda for sustainable development*.^1^ The SDGs are based on the principle of advancing equity and leaving no one behind in the process of economic, social and environmental development. The health-related targets of the SDG agenda, emphasized under SDG 3, reiterate this call for equity, with the overarching aim of ensuring healthy lives and promoting well-being for all at all ages. The SDGs require concerted efforts across diverse stakeholders within and outside the health sector to achieve improvements in the many conditions that affect health and the opportunity for health, such as poverty, gender discrimination, lack of educational opportunities, degradation of the natural environment and poor working conditions.

As countries work to improve health and its determinants in the SDG era, national monitoring of health inequalities will be a priority. Identifying health inequalities (differences in health across population subgroups) is an important part of addressing health inequities (differences in health that are deemed unfair or ethically problematic). Monitoring health inequalities helps countries to track progress towards SDG 3 and other health-related goals, and ensure that disadvantaged or hard-to-reach populations are not left behind.^2^^,^^3^ Thus, health inequality monitoring generates evidence to inform equity-oriented policies, programmes and practices that align with the intersectoral nature of the SDGs.^4^^,^^5^ For instance, progress towards universal health coverage – whereby people receive the health services they need without suffering financial hardship – requires monitoring of how essential health services are being expanded to serve the general public as well as the most disadvantaged population subgroups.^3^^,^^6^

Over the past decades, issues surrounding health inequality have gained attention in the domains of policy-making and academic research,^7^ initially in high-income countries, but increasingly too in low- and middle-income countries. The first widespread efforts to highlight social gradients in health emerged in the 1960s and 1970s in the United Kingdom of Great Britain and Northern Ireland. For example, the longitudinal Whitehall Studies began in the late 1960s, exploring inequalities in mortality among British civil servants by employment grade.^8^ Health inequalities gained traction in the political agendas of the 1980s and 1990s through the 1980 Black report, which was instrumental in bringing political attention, both nationally and internationally, to health inequalities in the United Kingdom.^9^ Subsequent reports reinforced the importance of addressing health inequalities.^10^^,^^11^ In particular, the final report of the Commission on Social Determinants of Health^12^ and other initiatives that emerged in the 2000s and 2010s^13^^,^^14^ brought renewed urgency to address health inequalities, now reflected in the 2030 agenda for sustainable development.^1^

The development of theoretical approaches and measurement techniques for health inequality monitoring largely stemmed from the fields of social epidemiology and health economics, with several key works published in the 1990s and 2000s.^15^^–^^19^ Contributions from other academic disciplines, including public health and the social sciences, continue to strengthen and shape current approaches to health inequality monitoring. Approaches that were primarily developed for use in high-income countries in Europe and North America have been adapted to be applicable across diverse geographical settings and in lower-income countries. 

The World Health Organization (WHO) has developed a multistep approach to monitoring health inequalities that can be applied at a national level to support SDG monitoring.^20^ The approach can also be applied at global or subnational levels, depending on the setting of interest. The five steps include: (i) determining the scope of monitoring for a given health topic, including relevant indicators of health and appropriate criteria for subgroup disaggregation (i.e. dimensions of inequality); (ii) obtaining the requisite data; (iii) analysing the data through disaggregation and the calculation of summary measures of inequality; (iv) reporting the results of monitoring to a defined target audience, show-casing relevant technical content in an appropriate and scientifically sound manner; and (v) using the evidence generated from health inequality monitoring as an input to decision-making about priority areas for further action. 

At each step of health inequality monitoring, there are multiple technical considerations that have implications for the conclusions and actions that stem from the monitoring process.^21^^,^^22^ The objective of this article is to present technical considerations for measuring health inequality, corresponding to aspects of selecting relevant dimensions of inequality (step i) and measuring and categorizing dimensions of inequality appropriately; disaggregating health data; and calculating summary measures of health inequality (step iii). Other considerations related to aspects of sourcing data (step ii), reporting results (steps iv) and implementing changes (step v) are discussed briefly.

## Measuring health inequalities

### Selecting dimensions of inequality

In the context of health inequality monitoring, a dimension of inequality refers to the broad criteria by which population subgroups are defined and health data are disaggregated. Dimensions of inequality can reflect social, economic, demographic, geographical and other characteristics by which health is unevenly distributed in a population. To give a more comprehensive picture of where health gaps exist within a population, robust health inequality monitoring involves measuring and reporting each health topic or indicator by multiple relevant dimensions of inequality. 

The selection of dimensions of inequality should include those that have widespread applicability, as well as those that are specifically relevant to the population being monitored.^23^ Stakeholders in the process should be consulted at the time of defining the monitoring framework to determine their interests and what key decisions are under consideration for particular strategies or programmes.

Under SDG 17 (strengthen the means of implementation), target 17.18 aims “to increase significantly the availability of high-quality, timely and reliable data.” The target calls for data to be disaggregated by “income, gender, age, race, ethnicity, migratory status, disability, geographic location and other characteristics relevant in national contexts.”^1^ Dimensions of inequality that are relevant to most settings, such as economic status, education, sex, age and place of residence, facilitate comparisons and benchmarking across populations (see the example from Indonesia in Box 1).

Box 1 **Dimensions of inequality and the subgroup categories applied to health inequality monitoring in Indonesia**

Economic status: quintile 1 (poorest); quintile 2; quintile 3; quintile 4; quintile 5 (richest).

Education: no education; primary school; secondary school or higher.

Occupation: employee; entrepreneur; farmer, fisherman or labourer; not working; other.

Employment status: not working; working.

Place of residence: rural; urban.

Age: < 1 year; 1–4 years; 5–14 years; 15–24 years; 25–34 years; 35–44 years; 45–54 years; 55–64 years; 65+ years.

Sex: female; male.

Subnational region: four provinces.

Source: *State of health inequality: Indonesia*, World Health Organization, 2017.^24^

Depending on the setting, other dimensions of inequality may be identified to shed light on unique situations and priorities within a given population. Ideally, the selection of relevant dimensions of inequality should draw from available evidence, such as reports or research findings, and reflect the social conditions, policies and other priorities within a population. For instance, caste is a relevant dimension of inequality in India,^25^^,^^26^ whereas race is particularly pertinent in the United States of America.^27^ In several European countries, migration status is an appropriate dimension of inequality.^28^ Another consideration when selecting relevant dimensions of inequality is the health topic or indicator. For example, a global report of inequality in childhood immunization revealed that many countries reported no difference in immunization coverage between boys and girls, whereas inequality in coverage based on maternal educational level was more pronounced. This finding suggests that maternal education may provide a strong basis for ongoing monitoring of equity in immunization coverage.^29^ Globally, age was reported to be a probable basis for discrimination in sexual and reproductive health, with adolescent girls tending to have worse outcomes than adult women in indicators such as contraceptive use.^30^

### Measuring dimensions of inequality

Different dimensions of inequality require different methods of measurement. The measurement of economic status, for instance, may be done using a household wealth index, individual income or household expenditure. Education may be measured by the total years of education or the highest level of education attained. The choice of measurement approach should reflect the context of the population and may also be aligned to support specific strategies or programmes. For example, in high-income countries, data about individual income may be an accurate depiction of economic status, as this is typically a readily-available figure that includes most (or all) sources of income, and that varies across the population. In many low- or middle-income countries, however, informal or non-cash economies may be more prevalent, and wealth indices (e.g. of asset ownership, housing conditions or access to services) may be a more appropriate measure.^16^ Previous analyses have demonstrated how different measures of economic status, applied to the same health data, can result in different conclusions about inequality.^31^

### Categorizing dimensions of inequality

Categorizing dimensions of inequality entails deciding on the technical criteria that guide how a population is broken into subgroups and the number of subgroups that are formed (Box 1). Economic status, for example, is most commonly presented as quintiles or sometime as deciles or two subgroups above or below a defined poverty threshold. Education may be presented using established categories such as primary school, secondary school and post-secondary education. Both education and age dimensions of inequality may be presented using defined ranges (e.g. 10-year age brackets) and variable numbers of subgroup categories. The use of a larger number of subgroups facilitates the exploration of characteristic patterns of inequality across subgroups ranked by a logical ordering (e.g. gradients). However, applying a larger number of subgroups may lead to more pronounced inequality between the extreme subgroups. The use of two subgroups may be useful to illustrate inequalities based on common demarcations within a population (e.g. between the poorest 40% versus the richest 60%,^32^ or between adolescents versus adults).

### Disaggregating data

A key step of health inequality monitoring involves breaking down the health indicator data to reflect the situation in each population subgroup, a process known as disaggregation. Disaggregated health data are useful to explore which population subgroups have a better or worse health situation and allow for the comparison of health indicator performance gaps between subgroups. In the context of the 2030 agenda for sustainable development, disaggregating data permits tracking progress in the achievement of the health targets among disadvantaged subgroups.^1^

The scope of disaggregation depends on the monitoring objective. Sometimes it is appropriate to disaggregate data by two or more dimensions of inequality, which permits the combined effects of different dimensions to be explored.^20^ The double or multiple disaggregation of health data can help to identify and draw attention to disadvantaged subgroups whose situation might otherwise be masked within a larger subgroup. For instance, double-disaggregation of data by place of residence and economic status illustrates the situation in the urban poor, a subgroup that is often especially vulnerable in countries that are experiencing rapid urbanization.^33^ In another example, in middle-income eastern European countries, double disaggregation of smoking prevalence by sex and socioeconomic status revealed opposite patterns in men (higher prevalence among the poorer) and women (higher prevalence among the richer).^34^ SDG 5, which addresses gender inequality, supports efforts to double disaggregate by sex, on top of other factors such as place of residence, to generate more gender-sensitive data to inform approaches to improve health.

### Calculating summary measures

Summary measures of health inequality draw from disaggregated data in two or more subgroups to yield a single number that represents inequality and are useful to make comparisons between health indicators and over time (see the example from Indonesia in Table 1). Broadly, summary measures of inequality reflect either absolute or relative inequality. Absolute inequality measures (e.g. difference) indicate the magnitude of difference in health between subgroups, and are easy to understand because they normally retain the same unit of measure as the health indicator. Relative inequality measures (e.g. ratio) show the proportional difference in health between subgroups, and because they are unitless they can be used to compare indicators with different units. Robust health inequality analyses involve the calculation of both absolute and relative summary measures.^29^^,^^35^

**Table 1:** Summary measures of inequality applied to calculate health inequalities in Indonesia

Summary measure	Description	Examples of relevant dimensions of inequality
Difference	Shows the absolute inequality between two subgroups: the mean value of a health indicator in one subgroup is subtracted from the mean value of that health indicator in another subgroup	Economic status education; occupation; employment status; place of residence; age; sex; subnational region
Ratio	Shows the relative inequality between two subgroups: the mean value of a health indicator in one subgroup is divided by the mean value of that health indicator in another subgroup	Economic status; education; occupation; employment status; place of residence; age; sex; subnational region
Mean difference from mean	Shows the difference, on average, of each subgroup from the population mean value; suitable for non-ordered dimensions with more than two subgroups	Occupation; subnational region
Index of disparity	Shows the mean difference from the mean value (above) expressed as a percentage of the overall mean; suitable for non-ordered dimensions with more than two subgroups	Occupation; subnational region
Slope index of inequality	Shows the absolute difference in predicted values of a health indicator between those that are the most advantaged (e.g. richest or most-educated subgroup) and those that are the most disadvantaged (e.g. the poorest or least-educated subgroup); suitable for ordered dimensions with more than two subgroups	Economic status; education
Relative index of inequality	Shows the relative difference in predicted values of a health indicator between those that are the most advantaged (e.g. richest or most-educated subgroup) and those that are the most disadvantaged (e.g. the poorest or least-educated subgroup); suitable for ordered dimensions with more than two subgroups	Economic status; education

Note: Adapted from *State of health inequality: Indonesia*, World Health Organization, 2017.^24^

A preliminary key consideration in calculating summary measures is whether to calculate simple summary measures only, or both simple and complex measures. Simple measures of inequality, such as difference or ratio, make pairwise comparisons between two subgroups, and can be calculated for any dimension of inequality, regardless of the number of subgroups. If a dimension of inequality has a natural ordering, such as economic status or education level, simple measures are typically calculated based on the two extreme subgroups (e.g. between richest and poorest quintiles, or between highest and lowest educational level). If a dimension of inequality does not have a natural ordering, such as geographical area or ethnicity, simple measures may be calculated using the two subgroups with the most extreme values. In some cases, it may be appropriate to calculate simple measures between the 95th and 5th percentiles (e.g. of subnational districts) or top and bottom deciles. Another option is to select one subgroup to serve as a reference, and compare all other subgroups to the reference. For example, for the ethnicity dimension of inequality, the majority subgroup might serve as a reference group, and difference and ratio measures can be calculated for each minority subgroup.

Complex measures of inequality make use of data from all subgroups, and can be calculated for dimensions with more than two subgroups. These measures overcome certain limitations of simple summary measures because they can take into account all subgroups, and the population share that belongs to each subgroup. However, they are inherently more difficult to comprehend because they combine many pieces of information in one single number and often make use of complex calculations. There are many different complex measures of inequality, each of which has different applicability depending on the characteristics of the underlying disaggregated data.^23^^,^^36^ For example, for ordered dimensions of inequality, the slope index of inequality and relative index of inequality are analogous to difference and ratio measures, respectively, and can be interpreted in a similar manner. Additionally, concentration index, a relative measure of inequality that has also absolute variant, is commonly applied in health economics to show the extent to which a health indicator is concentrated among the disadvantaged or the advantaged. For non-ordered dimensions of inequality, mean difference from mean (absolute measure) and the index of disparity (relative measure) are commonly applied. These may be calculated as weighted or unweighted measures, accounting for population share, or not, respectively. A key consideration of these non-ordered dimensions of inequality is the selection of a reference point against which the other subgroups are compared. For example, the reference point may be a selected reference subgroup, the best-performing subgroup or the national average.

## Other considerations

### Obtaining data

When a lack of data poses limitations on a country’s ability to monitor health inequality, efforts may be required to establish, strengthen or expand data collection practices. There are numerous tools available to support data collection systems. For example, household surveys, such as demographic and health surveys, multiple indicator cluster surveys and the WHO study on global ageing and adult health, are often deployed in low-resource settings to gather nationally representative data about specific health topics.^37^^–^^39^ Tools that support the regular collection of data from health facilities such as District Health Information System 2 platform^40^ and the WHO Service Availability and Readiness Assessment tool are used for routine collection of data from health facilities.^41^ Administrative data, civil registration and vital statistics, surveillance systems and national censuses are other important sources of data for health inequality monitoring.^23^ Data linkage can also be considered to fill in data gaps.^42^

### Reporting results

Regardless of its application, reporting should always be tailored to the target audience, and have a clear purpose and scope. There are special considerations, however, for health inequality reporting. A high-quality health inequality report needs to be transparent about the process of analysis and the assumptions that underlie the results and conclusions. For example, the rationale for the selection of relevant dimensions of inequality and their measurement and categorization needs to be clearly explained and justified. The presentation of numerous disaggregated data estimates and summary measures is common in health inequality monitoring reports, which may necessitate the use of figures or tables to concisely display results. The technical content of reports should provide sufficient information to give a complete and straightforward overview of health and health inequalities, enabling the reader to understand how the conclusions of the report were reached.

### Implementing changes

The results of health inequality monitoring can be applied to drive equity-oriented policies and programmes.^43^ Health inequality monitoring brings awareness as to how different subgroups of the population experience health, and can help to expose forms of disadvantage.^44^ Thus, implementing equity-oriented change may require action across both the health and non-health sectors and involve multiple stakeholders. The results of health inequality monitoring may serve as a basis for further investigation to determine the root causes of inequity and feasible approaches to address them.

## Conclusion

High-quality data that can be disaggregated and are timely, transparent and useable are key for the promotion of sustainable development.^45^ The technical aspects of measuring health inequalities warrant due consideration to ensure that monitoring is relevant to the underlying population, and that conclusions are based on sound, transparent analysis.

## References

[1] Resolution A/RES/70/1. Transforming our world: the 2030 agenda for sustainable development. In: Seventieth United Nations General Assembly, New York, 25 September 2015. New York: United Nations; 2015. Available from: http://www.un.org/ga/search/view_doc.asp?symbol=A/RES/70/1&Lang=E [cited 2018 May 1].

[2] Hosseinpoor AR, Bergen N, Magar V. Monitoring inequality: an emerging priority for health post-2015. Bull World Health Organ. 2015 9 1;93(9):591–591A. 10.2471/BLT.15.162081 26478619 PMC4581651

[3] World health statistics: monitoring health for the SDGs. Geneva: World Health Organization; 2017.

[4] Innov8 approach for reviewing national health programmes to leave no one behind: technical handbook. Geneva: World Health Organization; 2016.

[5] Petticrew M, Tugwell P, Welch V, Ueffing E, Kristjansson E, Armstrong R, et al. Better evidence about wicked issues in tackling health inequities. J Public Health (Oxf). 2009 9;31(3):453–6. 10.1093/pubmed/fdp076 19638396

[6] Hosseinpoor AR, Bergen N, Koller T, Prasad A, Schlotheuber A, Valentine N, et al. Equity-oriented monitoring in the context of universal health coverage. PLoS Med. 2014 9 22;11(9):e1001727. 10.1371/journal.pmed.1001727 25243463 PMC4171107

[7] Grove J, Claeson M, Bryce J, Amouzou A, Boerma T, Waiswa P, et al.; Kirkland Group. Maternal, newborn, and child health and the Sustainable Development Goals – a call for sustained and improved measurement. Lancet. 2015 10 17;386(10003):1511–4. 10.1016/S0140-6736(15)00517-6 26530604 PMC7613198

[8] Marmot MG, Rose G, Shipley M, Hamilton PJ. Employment grade and coronary heart disease in British civil servants. J Epidemiol Community Health. 1978 12;32(4):244–9. 10.1136/jech.32.4.244 744814 PMC1060958

[9] Black D, Morris JN, Smith C, Townsend P. The Black report. London: Department of Health and Social Security; 1980.

[10] Acheson D. Inequalities in health: report of an independent inquiry. London: Her Majesty’s Stationery Office; 1998.

[11] The Marmot review. Fair society, healthy lives. London: Institute for Health Equity; 2010. Available from: http://www.instituteofhealthequity.org/resources-reports/fair-society-healthy-lives-the-marmot-review/fair-society-healthy-lives-full-report-pdf.pdf [cited 2017 Sep 23].

[12] Commission on Social Determinants of Health. Closing the gap in a generation: health equity through action on the social determinants of health: final report of the commission on social determinants of health. Geneva: World Health Organization; 2008.

[13] Victora C, Requejo J, Boerma T, Amouzou A, Bhutta ZA, Black RE, et al. Countdown to 2030 for reproductive, maternal, newborn, child, and adolescent health and nutrition. Lancet Glob Health. 2017;4(11):e775–6. 10.1016/S2214-109X(16)30204-2 27650656

[14] Global Health Observatory data: health equity monitor [internet]. Geneva: World Health Organization; 2018. Available from: http://www.who.int/gho/health_equity/en/ [cited 2018 April 4]

[15] Anand S, Peter F, Sen A. Public health, ethics, and equity. Oxford: Oxford University Press; 2004.

[16] O’Donnell O, Van Doorslaer E, Wagstaff A, Lindelow M. Analyzing health equity using household survey data. Washington: World Bank; 2008.

[17] Mackenbach JP, Kunst AE. Measuring the magnitude of socio-economic inequalities in health: an overview of available measures illustrated with two examples from Europe. Soc Sci Med. 1997 3;44(6):757–71. 10.1016/S0277-9536(96)00073-1 9080560

[18] Wagstaff A, Paci P, van Doorslaer E. On the measurement of inequalities in health. Soc Sci Med. 1991;33(5):545–57. 10.1016/0277-9536(91)90212-U 1962226

[19] Gakidou E, Murray CJL, Frenk J. A framework for measuring health inequality. In: Murray CJL, Evans DB, editors. Health systems performance assessment: debates, methods and empiricism. Geneva: World Health Organization; 2003.

[20] National health inequality monitoring: a step-by-step manual. Geneva: World Health Organization; 2017.

[21] Harper S, King NB, Meersman SC, Reichman ME, Breen N, Lynch J. Implicit value judgments in the measurement of health inequalities. Milbank Q. 2010 3;88(1):4–29. 10.1111/j.1468-0009.2010.00587.x 20377756 PMC2888011

[22] Kjellsson G, Gerdtham U-G, Petrie D. Lies, damned lies, and health inequality measurements: understanding the value judgments. Epidemiology. 2015 9;26(5):673–80. 10.1097/EDE.0000000000000319 26133019 PMC4521896

[23] Handbook on health inequality monitoring: with a special focus on low-and middle-income countries. Geneva: World Health Organization; 2013.

[24] State of health inequality: Indonesia. Geneva: World Health Organization; 2017.

[25] Desai S, Dubey A. Caste in 21st Century India: Competing Narratives. Econ Polit Wkly. 2012 3 12;46(11):40–9. 22736803 PMC3379882

[26] India National Family Health Survey 2015–2016. Mumbai: International Institute for Population Sciences; 2017.

[27] Race [internet]. Washington: United States Census Bureau; 2018. Available from: https://www.census.gov/topics/population/race/about.html [cited 2018 June 25].

[28] European Network of Legal Experts in the nondiscrimination field. Links between migration and discrimination. Luxembourg: European Commission; 2009.

[29] State of inequality: childhood immunization. Geneva: World Health Organization; 2016.

[30] Morris JL, Rushwan H. Adolescent sexual and reproductive health: the global challenges. Int J Gynaecol Obstet. 2015 10;131 Suppl 1:S40–2. 10.1016/j.ijgo.2015.02.006 26433504

[31] Lindelow M. Sometimes more equal than others: how health inequalities depend on the choice of welfare indicator. Health Econ. 2006 3;15(3):263–79. 10.1002/hec.1058 16259049

[32] Poverty and shared prosperity 2016: taking on Inequality. Washington: World Bank; 2016.

[33] Global report on urban health: equitable, healthier cities for sustainable development. Geneva: World Health Organization; 2016. Available from: http://apps.who.int/iris/bitstream/10665/204715/1/9789241565271_eng.pdf?ua=1 [cited 2018 Jun 12].

[34] Hosseinpoor AR, Parker LA, Tursan d’Espaignet E, Chatterji S. Socioeconomic inequality in smoking in low-income and middle-income countries: results from the World Health Survey. PLoS One. 2012;7(8):e42843. 10.1371/journal.pone.0042843 22952617 PMC3430638

[35] State of inequality: reproductive, maternal, newborn and child health. Geneva: World Health Organization; 2015.

[36] Health equity assessment toolkit plus upload database edition: technical notes. Geneva: World Health Organization; 2016.

[37] The DHS program: demographic and health surveys [internet]. Washington: United States Agency for International Development; 2018. Available from: http://dhsprogram.com [cited 2018 May 21].

[38] Multiple indicator cluster surveys [internet]. New York: United Nations Children’s Fund; 2018. Available from: mics.unicef.org [cited 2018 May 21].

[39] WHO study on global AGEing and adult health (SAGE) [internet]. Geneva: World Health Organization; 2018. Available from: http://www.who.int/healthinfo/sage/en/ [cited 2018 May 21].

[40] DHIS2 [internet]. Oslo: Health Information Systems Programme; 2018. Available from: https://www.dhis2.org/ [cited 2018 May 21].

[41] Service availability and readiness assessment (SARA) [internet]. Geneva: World Health Organization; 2018. Available from: http://www.who.int/healthinfo/systems/sara_introduction/en/ [cited 2018 May 21].

[42] Hosseinpoor AR, Nambiar D, Suparmi, Kusumawardani N. Data source mapping: an essential step for health inequality monitoring. Glob Health Action. 2018;11 sup1:1456743. 10.1080/16549716.2018.1456743 29768133 PMC5965029

[43] Koller TS, Saint V, Floranita R, Koemara Sakti GM, Pambudi I, Hermawan L, et al. Applying the Innov8 approach for reviewing national health programmes to leave no one behind: lessons learnt from Indonesia. Glob Health Action. 2018 Jan-Dec;11 sup1:1423744. 10.1080/16549716.2018.1423744 29569529 PMC5912432

[44] Exploration of inequality: childhood immunization. Geneva: World Health Organization; 2018 [in press].

[45] Independent Expert Advisory Group on a Data Revolution for Sustainable Development. A world that counts: mobilizing the data revolution for sustainable development. New York: United Nations; 2014.

